# Beta-Blocker Use After Acute Myocardial Infarction: Differential Impact Across Heart Failure Subtypes in a Real-World Cohort

**DOI:** 10.1016/j.jacadv.2026.103168

**Published:** 2026-08-19

**Authors:** Tianshu Gu, Sutao Hu, Yukun Zhang, Wanlin Feng, Yongjian Li, Xing Liu, Xian Shao, Lin Wang, Tong Liu, Gregory Y.H. Lip, Kang-Yin Chen

**Affiliations:** a Tianjin Key Laboratory of Ionic-Molecular Function of Cardiovascular disease, Department of Cardiology, Tianjin Institute of Cardiology, The Second Hospital of Tianjin Medical University, Tianjin, China; b Department of Cardiology, Tianjin Nankai Hospital, Tianjin Medical University, Tianjin, China; c Division of Nephrology, National Clinical Research Center for Kidney Disease, State Key Laboratory of Organ Failure Research, Nanfang Hospital, Southern Medical University, Guangzhou, China; d Department of Cardiology, Chest Hospital of Tianjin University, Tianjin, China; e Liverpool Centre for Cardiovascular Science at University of Liverpool, Liverpool John Moores University and Liverpool Heart & Chest Hospital, Liverpool, United Kingdom; f Department of Clinical Medicine, Aalborg University, Aalborg, Denmark; g Department of Cardiology, Lipidology and Internal Medicine with Intensive Coronary Care Unit, Medical University of Bialystok, Bialystok, Poland

**Keywords:** acute myocardial infarction, beta-blocker, heart failure, real-world study

## Abstract

**Background:**

Beta-blocker (BB) benefits after acute myocardial infarction (AMI) may differ by heart failure (HF) phenotype in the contemporary reperfusion era.

**Objectives:**

The authors evaluated associations of in-hospital BB therapy with 1-year outcomes after AMI complicated by HF, stratified by left ventricular ejection fraction phenotype.

**Methods:**

We analyzed 5,557 patients with AMI complicated by HF from the Tianjin Coronary Artery Disease Specialized Database, including 3,962/5,557 (71.3%) with HF with preserved ejection fraction (HFpEF) and 1,595/5,557 (28.7%) with HF with reduced or mildly reduced ejection fraction (HFrEF/HFmrEF). The primary endpoint was a 1-year composite of cardiac death or HF rehospitalization. We assessed associations using inverse probability of treatment weighting, Cox models, and competing risk analyses. Sensitivity analyses comprised propensity score matching, win ratio, and time-stratified Cox models.

**Results:**

Overall, 859/5,557 patients (15.5%; 95% CI: 14.5%-16.4%) reached the primary endpoint by 1 year; median follow-up was 1,566 days (IQR: 882-2,354). Interaction by HF phenotype was significant (*P* = 0.034). After adjustment, BB therapy was associated with lower primary endpoint risk in HFrEF/HFmrEF (adjusted HR [aHR]: 0.590; 95% CI: 0.438-0.795; *P* < 0.001), but not in HFpEF. BB therapy was also associated with lower risks of all-cause death (aHR: 0.577; 95% CI: 0.395-0.841; *P* = 0.004) and cardiac death (aHR: 0.536; 95% CI: 0.349-0.823; *P* = 0.004) in HFrEF/HFmrEF. Sensitivity analyses yielded consistent findings.

**Conclusions:**

Among patients with AMI complicated by HF, in-hospital BB use was associated with lower 1-year adverse outcomes risk in HFrEF/HFmrEF, but not in HFpEF.

Heart failure (HF) remains the leading cause of hospitalization in the contemporary era, predominantly driven by acute myocardial infarction (AMI). Patients with AMI, particularly those who develop left ventricular systolic dysfunction or symptoms of congestion, are at high risk for long-term adverse cardiovascular outcomes, including HF rehospitalization and death.^1^ Despite advances in the management of AMI, HF remains a highly prevalent complication.^2^^,^^3^ Key interventions, encompassing early reperfusion and therapies aimed at reversing myocardial remodeling—such as beta-blockers (BBs) and renin-angiotensin inhibitors (RASi)—have markedly improved outcomes in AMI.^4^ Nonetheless, a substantial residual risk of post-MI HF and other adverse outcomes persists in this patient population.

Studies on trends in guideline-directed medical therapy utilization for HF reveal that BBs are the most frequently prescribed component across all HF phenotypes, including HF with preserved ejection fraction (HFpEF), HF with reduced ejection fraction (HFrEF), and HF with mildly reduced ejection fraction (HFmrEF).^5^ Nevertheless, contemporary acute coronary syndrome (ACS) management guidelines uniformly acknowledge heterogeneity in evidence supporting BB use in AMI patients without reduced EF or overt HF.^6^^,^^7^ Consequently, these guidelines issue distinct evidence-graded recommendations for BB use in HFrEF and HFmrEF patients post-MI but provide no specific recommendation for HFpEF. Two landmark randomized controlled trials (RCTs) recently challenged post-AMI BB use: The REDUCE-AMI (Randomized Evaluation of Decreased Usage of Beta-Blockers after Acute Myocardial Infarction) trial demonstrated that long-term BB therapy did not reduce the composite risk of recurrent MI or death in AMI patients with preserved left ventricular ejection fraction (LVEF) (≥50%).^8^ Conversely, the ABYSS (Beta-Blocker Interruption After Uncomplicated Myocardial Infarction) trial found that although discontinuing long-term BB therapy in post-MI patients with LVEF >40% met noninferiority criteria compared to continued treatment, it was associated with numerically higher rates of major adverse outcomes (death, MI, stroke, or cardiovascular hospitalization).^9^

These landmark trials provide crucial evidence for BB strategies in post-MI patients defined primarily by LVEF thresholds. Addressing a distinct clinical scenario, our study investigates real-world AMI patients across different LVEF spectrums who concurrently present with overt HF symptoms or biomarker-based evidence of congestion. We therefore used a real-world cohort to examine whether the association between in-hospital BB use and 1-year outcomes differs across HF subtypes among patients with AMI.

## Methods

### Data sources

The data for this study were sourced from the Tianjin Coronary Artery Disease (CAD) Specialized Database. The details of the platform have been described in our previous publications.^10^^,^^11^ Briefly, the CAD specialized database includes patients who were hospitalized at least once between January 1, 2010, and March 31, 2024, with discharge diagnoses including CAD. All health care information for these patients is collected, including demographic characteristics, disease diagnoses, medication and nonmedication prescriptions, examination and laboratory test results, surgical information, cost details, community medication and health examination information, and public health mortality information. The International Classification of Diseases-10th Revision codes used to define diagnostic variables are listed in Supplemental Table 1. All exposure and outcome variables were subjected to manual sampling cross-verification between our CAD specialized database records and source medical documentation to ensure data authenticity.^12^ A total of 100 patient records were included in this manual cross-verification. The study protocol adhered to the ethical principles of the 1975 Declaration of Helsinki and received prior approval (KY2023052-01) from the Clinical Research Ethics Committee of Tianjin Medical University Second Hospital, with a waiver of informed consent due to the retrospective nature of data analysis.

### Study population and outcomes

We included adult patients (≥18 years) presenting with AMI complicated by HF, defined as Killip class >I or Killip class I with elevated N-terminal pro-B-type natriuretic peptide (NT-proBNP) levels. Given the dynamic behavior of NT-proBNP in the AMI setting, we utilized the initial baseline NT-proBNP value measured within 24 hours of hospital admission. To define HF in Killip class I patients, we applied age-stratified diagnostic cutoffs: >450 pg/mL for patients aged <50 years, >900 pg/mL for ages 50 to 75 years, and >1,800 pg/mL for patients >75 years.^13^ A complete case analysis approach was adopted for this inclusion criterion; patients with missing baseline NT-proBNP data were excluded. Because NT-proBNP was used solely for eligibility assessment, patients with and without available NT-proBNP measurements were compared across observed baseline characteristics. All participants underwent transthoracic echocardiography during index hospitalization. LVEF-based HF phenotyping was performed according to the 2021 European Society of Cardiology guidelines for HF (HFrEF: LVEF <40%, HFmrEF: LVEF 40% to 49%, HFpEF: LVEF ≥50%).^14^ In this AMI setting, the HFpEF category denotes patients with preserved EF and concurrent clinical or biomarker evidence of HF during the index hospitalization, rather than implying that AMI directly caused a de novo HFpEF syndrome. Although patients with documented prior HF were excluded, HFpEF in this context may still reflect unmasking or decompensation of pre-existing, often subclinical, diastolic, or structural heart disease. Considering the impact of sample size on statistical power and the clinical characteristics of the patients, we combined HFrEF and HFmrEF into a single group for the primary analysis. This approach is supported by previous studies that have demonstrated the clinical similarities between these 2 HF phenotypes,^15^^,^^16^ and a prespecified exploratory analysis stratified by LVEF <40% vs 40% to 49% confirmed consistent effects. Patients were excluded based on the following criteria: 1) age <18 years; 2) prior history of HF; 3) contraindications to BB therapy (including asthma, sinus arrest, sick sinus syndrome, sinus bradycardia, or atrioventricular block); 4) in-hospital mortality (to mitigate immortal time bias); 5) missing echocardiography or NT-proBNP data; and 6) LVEF ≥50% without concurrent HF symptoms or congestion biomarkers (Supplemental Figure 1).

The primary endpoint was a composite of cardiac death and HF-related rehospitalization within 1 year. HF-related rehospitalization was defined as hospitalization primarily due to worsening HF, hospitalization for other causes but with concurrent HF decompensation at admission, or hospitalization for other causes complicated by HF worsening during the hospital stay. Secondary endpoints included the individual components of the primary endpoint and all-cause mortality. Bleeding events were defined as gastrointestinal, intracranial, or other major bleeding, such as ocular or respiratory hemorrhage.^17^ Follow-up began on the date of discharge from the index AMI hospitalization. Patients were followed until the first occurrence of the outcome of interest, the last available health care record, or 365 days after discharge, whichever occurred first.

### Statistical analysis

Our research methods were conducted in strict compliance with the STROBE (Strengthening the Reporting of Observational Studies in Epidemiology) guidelines. Patients were categorized into 2 groups based on in-hospital BB use: those who were prescribed long-term BB medical orders during hospitalization and those who did not receive BB therapy during the index admission. Preadmission BB use was ascertained from community/outpatient medication records preceding the index hospitalization. Analyses were performed separately within the HFpEF cohort and the combined HFrEF/HFmrEF cohort. In the description of 2 cohorts’ patient characteristics according to BBs use, continuous variables are described using mean with SD or median with IQR. Categorical variables are summarized by absolute and relative frequencies. Comparisons between groups were performed using a *t*-test or Mann-Whitney *U* test for continuous variables and Fisher exact test or chi-square test for categorical variables, as appropriate. To formally assess whether the association between BB use and the primary outcome differed by HF phenotype, a multiplicative interaction term between BB use and HF phenotype (HFpEF vs HFrEF/HFmrEF) was included in the unweighted multivariable Cox model.

First, inverse probability treatment weighting (IPTW) was applied to minimize confounding between patients who did and did not receive BB therapy in the real-world setting. Variables used to estimate the weights were selected based on both intergroup baseline imbalances and clinical relevance. A directed acyclic graph describes the causal structure between study exposure, outcomes, and confounders in Supplemental Figure 2. Specifically, propensity scores (PSs) for in-hospital BB use were estimated separately in the HFpEF and HFrEF/HFmrEF cohorts using multivariable logistic regression including age, sex, Killip class, percutaneous coronary intervention (PCI), diabetes mellitus, atrial fibrillation, hypertension, cerebrovascular disease, aspirin, P2Y12 inhibitors, statins, mineralocorticoid receptor antagonists (MRAs), renin–angiotensin system inhibitors, and sodium glucose co-transporter 2 inhibitors (SGLT2i). Stabilized inverse probability weights targeting the average treatment effect were calculated as the marginal probability of the observed treatment divided by its PS-based conditional probability. Weight distributions were assessed using summary statistics and visual inspection, with extreme values truncated at the 1st and 99th percentiles. An absolute standardized mean differences < 0.10 was considered to indicate adequate covariate balance (Supplemental Figure 3).^18^^,^^19^

Time-to-event outcomes were illustrated using weighted Kaplan-Meier cumulative incidence curves. Both the primary endpoint and secondary outcomes were analyzed using multivariable Cox proportional hazards models. The proportional hazards assumption for the IPTW with additional covariate adjustment Cox models was assessed using Schoenfeld residuals. No significant violations were identified for BB use or in the global model tests. Three sequential multivariable models were constructed with increasing levels of covariate adjustment. Model 1 included BB use, sex, age, in-hospital frailty levels,^20^ Killip class, and AMI type (ST-segment elevation myocardial infarction [STEMI] or non–STEMI). Model 2 further adjusted for comorbidities, including hypertension, diabetes mellitus, atrial fibrillation, cerebrovascular disease, and peripheral artery disease. Model 3 additionally incorporated in-hospital medication use: aspirin, P2Y12 inhibitors, MRAs, SGLT2i, RASi, calcium-channel blockers (CCBs), and statins. Outcomes other than mortality were analyzed using Fine–Gray models to account for the competing risk of death. IPTW Cox models and Fine–Gray models were fitted within the weighted pseudopopulations, with models 1 to 3 additionally incorporating sequential covariate adjustment to address any residual imbalance persisting after weighting and to assess the robustness of the estimates across different levels of adjustment. An unweighted multivariable Cox model incorporating the full covariate set (equivalent to model 3) was additionally fitted as a reference to contextualize the effect of IPTW. Restricted cubic spline (RCS) analyses were conducted to further evaluate the association and potential nonlinearity between LVEF and the primary outcome in both the post-AMI HFpEF cohort and the post-AMI HFrEF/HFmrEF cohort. For all RCS analyses, 4 knots were prespecified following Harrell’s recommendations and placed at the 5th, 35th, 65th, and 95th percentiles of the LVEF distribution in the corresponding analytic sample.

Subgroup analyses were performed in the 2 cohorts to explore the consistency of treatment effects across clinically relevant categories, including sex, frailty levels, type of AMI, presence of hypertension, diabetes mellitus, or atrial fibrillation, use of RASi, MRA, and SGLT2i, and whether the patient underwent PCI. Furthermore, an exploratory analysis was conducted within the HFrEF/HFmrEF cohort to evaluate the consistency of BB effects across the HFrEF and HFmrEF subgroups, with interaction testing applied to assess potential effect modification. To ensure the robustness of the primary findings, several sensitivity analyses were conducted. First, PS–adjusted Cox regression models were separately applied within the AMI–HFpEF and AMI–HFrEF/HFmrEF cohorts. Second, 1:1 nearest-neighbor PS matching (PSM) without replacement was performed in both populations using a caliper width of 0.02. Cox proportional hazards models stratified by matched pair were then used to evaluate the association between in-hospital BB use and the primary outcome in the matched populations. Given that baseline characteristics were well balanced after PSM, we further used the win ratio method in both matched subgroups, with cardiac death prioritized over HF-related rehospitalization.^21^^,^^22^ To address the potential mixing of continuation of preadmission BB therapy and new in-hospital BB use, we performed an additional sensitivity analysis after excluding patients with documented preadmission BB use. Finally, to account for temporal variations in clinical practice, a time-stratified analysis was performed. The study period was divided into 2 epochs (2012-2017 and 2018-2023), and Cox models were fitted separately in each period within both HF subgroups using the IPTW-adjusted data set.

Two-sided *P* values of <0.05 were considered statistically significant. The statistical tests were performed using R programming version 4.4.1 (The R Foundation for Statistical Computing).

## Results

### Baseline characteristics

Of the 163,335 patients with first-time AMI identified from the Tianjin CAD Specialized Database, 5,557 met the prespecified inclusion and exclusion criteria and comprised the final study cohort (Supplemental Figure 1). Among all 5,557 included patients, 859 experienced the primary outcome within 1 year, corresponding to an overall event rate of 15.5% (95% CI: 14.5%-16.4%). The median overall available follow-up was 1,566 days (IQR: 882-2,354 days). No statistically significant differences in observed baseline characteristics were identified between patients with and without available NT-proBNP measurements (Supplemental Table 2).

### Post-AMI HFpEF

Among patients with AMI and HFpEF, 3,331/3,962 (84.1%) received BBs during hospitalization (Table 1). Compared with those who did not receive BB therapy, patients in the BB group were more likely to be male (2,238/3,331 [67.2%] vs 396/631 [62.8%]; *P* = 0.034), were younger (64.85 ± 11.95 vs 68.73 ± 11.39; *P* < 0.001), and presented with lower Killip class and hospital frailty risk level. No significant differences were observed between groups in the proportion of STEMI or LVEF. Regarding comorbidities, the BB group had lower Charlson Comorbidity Index scores and was less likely to have peripheral artery disease, cerebrovascular disease, gastrointestinal bleeding, or diabetes mellitus. In terms of in-hospital treatments, the BB group was more likely to undergo PCI, MRAs, RASis, and statins. There were no significant differences in the use of loop diuretics, CCBs, or SGLT2i between the 2 groups (Table 1).

**Table 1:** Baseline Characteristics According to Beta-Blocker Use or Nonuse by Left Ventricular Ejection Fraction Phenotype

	Post-AMI HFpEF Population				Post-AMI HFrEF-HFmrEF Population			
	No BBs Group (n = 631)	BBs Group (n = 3,331)	SMD Before Weighting	SMD After Weighting	No BBs Group (n = 251)	BBs Group (n = 1,344)	SMD Before Weighting	SMD After Weighting
Male, n (%)	396 (62.8)	2,238 (67.2)	0.093	0.017	160 (63.7)	969 (72.1)	0.180	0.043
Age, y, mean (SD)	68.73 (11.39)	64.85 (11.95)	0.332	0.096	71.78 (11.51)	67.47 (12.17)	0.364	0.073
STEMI, n (%)	327 (51.8)	1,599 (48.0)	0.076	0.053	113 (45.0)	566 (42.1)	0.059	0.005
Killip class, n (%)			0.216	0.032			0.279	0.073
I	214 (33.9)	1,168 (35.1)			62 (24.7)	288 (21.4)		
II	297 (47.1)	1,781 (53.5)			97 (38.6)	693 (51.6)		
III	91 (14.4)	290 (8.7)			64 (25.5)	274 (20.4)		
IV	29 (4.6)	92 (2.8)			28 (11.2)	89 (6.6)		
Hospital frailty risk level			0.172	0.019			0.392	0.083
Low risk	353 (55.9)	2,111 (63.4)			91 (36.3)	740 (55.1)		
Intermediate	267 (42.3)	1,198 (36.0)			152 (60.6)	586 (43.6)		
High risk	11 (1.7)	22 (0.7)			8 (3.2)	18 (1.3)		
LVEF	60 [56, 62]	60 [56, 62]	0.053	0.027	41 [36, 45]	42 [38, 46]	0.214	0.092
NT-proBNP	799.25 [311.88, 2,510.50]	792.65 [326.15, 1,964.62]	0.160	0.029	5,693.00 [1,465.00, 13,400.00]	2,921.00 [902.15, 6,916.50]	0.452	0.086
Comorbidities								
CCI	1.00 [0.00, 2.00]	0.00 [0.00, 2.00]	0.116	0.037	1.00 [0.00, 3.00]	1.00 [0.00, 3.00]	0.201	0.032
Peripheral vascular disease	88 (13.9)	295 (8.9)	0.161	0.075	28 (11.2)	107 (8.0)	0.109	0.081
Cerebrovascular disease	180 (28.5)	804 (24.1)	0.100	0.008	90 (35.9)	371 (27.6)	0.178	0.051
Gastrointestinal bleeding	27 (4.3)	86 (2.6)	0.093	0.040	12 (4.8)	35 (2.6)	0.116	0.052
Diabetes mellitus	198 (31.4)	1,247 (37.4)	0.128	0.066	107 (42.6)	550 (40.9)	0.035	0.084
Malignancy	23 (3.6)	83 (2.5)	0.067	0.005	10 (4.0)	26 (1.9)	0.121	0.008
Atrial fibrillation	76 (12.0)	352 (10.6)	0.047	0.059	50 (19.9)	224 (16.7)	0.084	0.079
Hypertension	396 (62.8)	2,192 (65.8)	0.064	0.025	158 (62.9)	824 (61.3)	0.034	0.015
Medications								
PCI	312 (49.4)	2,279 (68.4)	0.393	0.060	89 (35.5)	676 (50.3)	0.303	0.003
Primary PCI	257 (40.7)	1,845 (55.4)	0.297	0.040	67 (26.7)	494 (36.8)	0.217	0.027
Aspirin	533 (84.5)	3,160 (94.9)	0.347	0.013	189 (75.3)	1,261 (93.8)	0.530	0.008
P2Y12 inhibitors	584 (92.6)	3,252 (97.6)	0.237	0.054	216 (86.1)	1,309 (97.4)	0.421	0.051
Statins	576 (91.3)	3,273 (98.3)	0.317	0.020	211 (84.1)	1,309 (97.4)	0.472	0.049
CCBs	138 (21.9)	636 (19.1)	0.069	0.027	40 (15.9)	173 (12.9)	0.087	0.059
MRAs	266 (42.2)	1,619 (48.6)	0.130	0.004	159 (63.3)	1,050 (78.1)	0.329	0.008
RASis	378 (59.9)	2,915 (87.5)	0.660	0.031	127 (50.6)	1,123 (83.6)	0.749	0.024
SGLT2 inhibitors	41 (6.5)	257 (7.7)	0.047	0.017	24 (9.6)	142 (10.6)	0.033	0.004
Loop diuretics	398 (63.1)	2,168 (65.1)	0.042	0.047	208 (82.9)	1,152 (85.7)	0.078	0.041
OACs	23 (3.6)	73 (2.2)	0.086	0.085	10 (4.0)	60 (4.5)	0.024	0.039
Preadmission BB use	184 (29.2)	1,062 (31.9)	0.059	0.010	85 (33.9)	491 (36.5)	0.056	0.021

Effective sample size after weighting: 470.0 and 3,188.3 in the no-beta-blocker and beta-blocker groups, respectively, for the HFpEF cohort; and 245.9 and 1,215.2, respectively, for the HFrEF/HFmrEF cohort.

AMI = acute myocardial infarction; BB = beta-blocker; CCBs = calcium-channel blockers; CCI = Charlson Comorbidity Index; HFmrEF = heart failure with mildly reduced ejection fraction; HFpEF = heart failure with preserved ejection fraction; HFrEF = heart failure with reduced ejection fraction; LVEF = left ventricular ejection fraction; MRAs = mineralocorticoid receptor antagonists; NT-proBNP = N-terminal pro-B-type natriuretic peptide; OAC = oral anticoagulant; PCI = percutaneous coronary intervention; RASi = renin-angiotensin inhibitor; SGLT2i = sodium glucose co-transporter 2 inhibitor; SMD = standardized mean difference; STEMI = ST-segment elevation myocardial infarction.

### Post-AMI HFrEF/HFmrEF

In the post-AMI HFrEF/HFmrEF population, 1,344/1,595 patients (84.3%) received BB therapy during hospitalization (Table 1). Like the HFpEF population, patients treated with BBs were younger (67.47 ± 12.17 vs 71.78 ± 11.5; *P* < 0.001), more likely to be male (969/1,344 [72.1%] vs 160/251 [63.7%]; *P* = 0.009), and had lower Killip class, Charlson Comorbidity Indexes, and hospital frailty level. The proportion of STEMI was comparable between groups. Regarding comorbid conditions, patients receiving BBs had a lower prevalence of cerebrovascular disease (371/1,344 [27.6%] vs 90/251 [35.9%]; *P* = 0.010). No significant differences were observed between groups in the prevalence of peripheral artery disease, gastrointestinal bleeding, diabetes mellitus, malignancy, atrial fibrillation, or hypertension. In-hospital treatments also showed similar patterns to those observed in the HFpEF group. Patients treated with BBs were more likely to undergo PCI and receive antiplatelet agents (all *P* < 0.001), MRAs, RASis, and statins. There were no significant differences in the use of loop diuretics, CCBs, or SGLT2i.

### Outcomes analysis

In the overall cohort, multiplicative interaction analysis using an unweighted multivariable Cox model demonstrated a significant interaction between in-hospital BB use and HF phenotype for the 1-year primary outcome (HR for interaction; 0.704; 95% CI: 0.509-0.974; *P* for interaction = 0.034). The association of BB use with the primary outcome differed between patients with HFpEF and those with HFrEF/HFmrEF, with a more favorable association in the latter group.

### Post-AMI HFpEF

**Figure 1:**
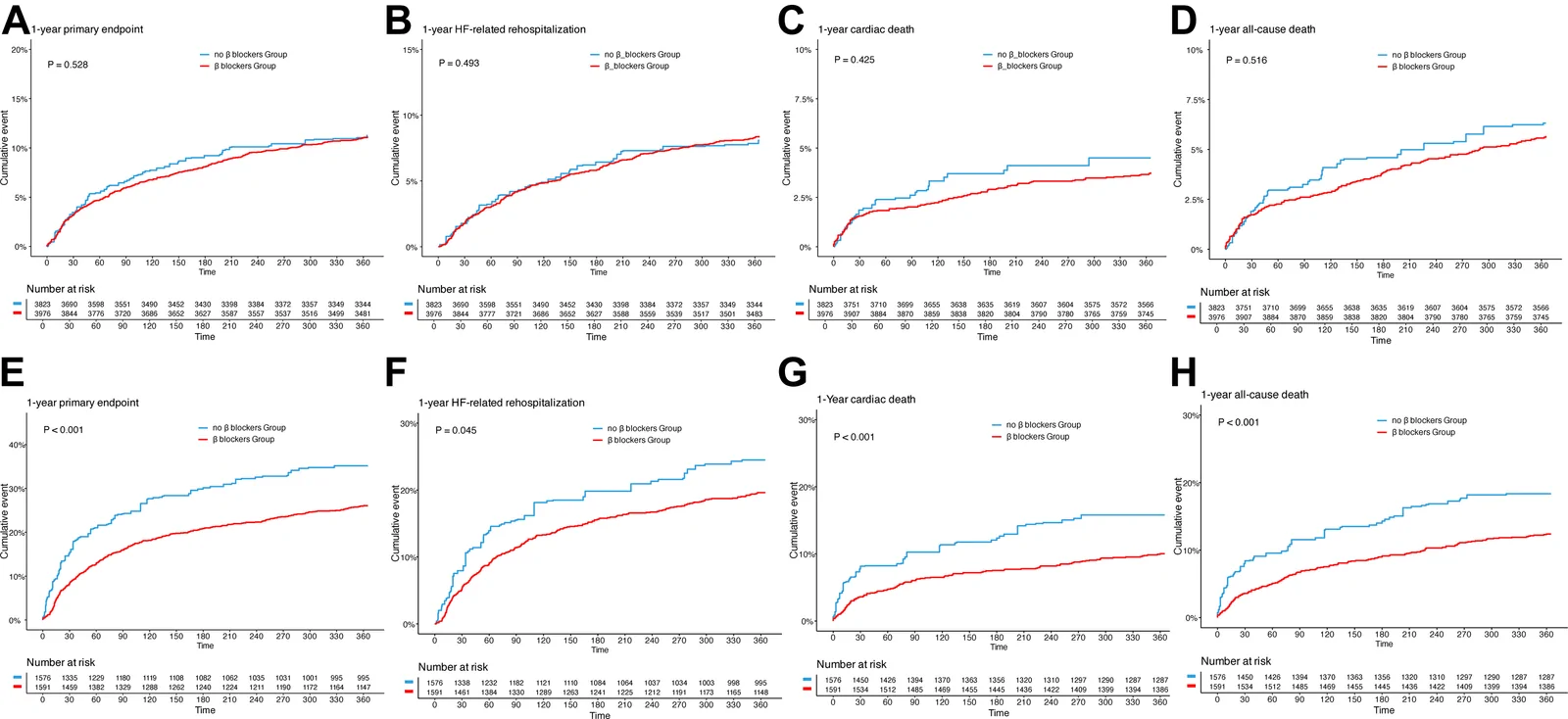
Kaplan-Meier Curves of Outcomes by Beta-Blocker Use Kaplan-Meier plots showing the cumulative incidence (%) of 1-year primary and secondary outcomes after acute myocardial infarction in patients with (red) vs without (blue) beta-blocker therapy, stratified by heart failure phenotype: post-acute myocardial infarction heart failure with preserved ejection fraction (A to D) and post-acute myocardial infarction heart failure with reduced ejection fraction/heart failure with mildly reduced ejection fraction (E to H). Primary outcome was defined as the composite outcome including cardiac death and heart failure–related rehospitalization. HF = heart failure.

After IPTW adjustment for baseline confounders, the standardized mean differences for all covariates were ≤0.10 (Supplemental Figure 3); in post-AMI HFpEF patients, the 1-year incidence of the primary outcome did not differ significantly between the 2 groups (Figures 1A to 1D). Both the unweighted multivariable Cox regression (adjusted HR [aHR], 1.016; 95% CI: 0.795-1.230; *P* = 0.897) and the IPTW Cox models with additional covariate adjustment (model 1: 0.997 [0.765-1.280], *P* = 0.980; model 2: 0.997 [0.767-1.296], *P* = 0.982; model 3: 1.054 [0.810-1.371], *P* = 0.694) demonstrated no significant difference between the groups. Similarly, for secondary outcomes at 1 year among post-AMI HFpEF patients, BB use was not significantly associated with all-cause mortality (aHR: 1.015; 95% CI: 0.738-1.397; *P* = 0.927), cardiac death (aHR: 1.000; 95% CI: 0.680-1.471; *P* = 0.999), or HF-related rehospitalization (aHR: 1.023; 95% CI: 0.753-1.390; *P* = 0.884). Three IPTW Cox models with additional covariate adjustment incorporating different covariates for further adjustment yielded consistent results (Table 2). To account for the competing risk of mortality, IPTW Fine-Gray models with additional covariate adjustment showed that in-hospital BBs use was not significantly associated with 1-year primary outcome risk across 3 adjusted models (model 1 subdistribution HR [sHR]: 0.831 [0.648-1.060], *P* = 0.140; model 2 sHR: 0.828 [0.646-1.060], *P* = 0.140; model 3 sHR: 1.020 [0.779-1.330], *P* = 0.910) (Table 3).

**Table 2:** Primary and Secondary Outcomes in Post-AMI HFpEF and HFrEF/HFmrEF Populations: Cox Models Before and After IPTW Adjustment

Unweighted Multivariable Cox Model	1-Year Primary Outcome		1-Year All-Cause Mortality		1-Year Cardiac Mortality		1-Year HF-Related Rehospitalization	
	aHR (95% CI)	*P* Value	aHR (95% CI)	*P* Value	aHR (95% CI)	*P* Value	aHR (95% CI)	*P* Value
Post-AMI HFpEF population								
	1.016 (0.795-1.230)	0.897	1.015 (0.738-1.397)	0.927	1.000 (0.680-1.471)	0.999	1.023 (0.753-1.390)	0.884
IPTW models with additional covariate adjustment								
Model 1	0.997 (0.765-1.280)	0.980	1.005 (0.695-1.453)	0.977	0.992 (0.643-1.531)	0.974	1.008 (0.732-1.388)	0.962
Model 2	0.997 (0.767-1.296)	0.982	1.014 (0.705-1.459)	0.937	1.001 (0.650-1.540)	0.997	0.988 (0.719-1.359)	0.943
Model 3	1.054 (0.810-1.371)	0.694	1.023 (0.705-1.486)	0.901	1.044 (0.676-1.611)	0.845	1.031 (0.748-1.420)	0.853
Post-AMI HFrEF/HFmrEF population								
	0.619 (0.483-0.792)	<0.001^a^	0.596 (0.431-0.826)	0.002^a^	0.580 (0.404-0.833)	0.003^a^	0.725 (0.527-0.998)	0.049^a^
IPTW models with additional covariate adjustment								
Model 1	0.646 (0.486-0.860)	0.003^a^	0.598 (0.416-0.860)	0.006^a^	0.564 (0.376-0.847)	0.006^a^	0.751 (0.522-1.079)	0.122
Model 2	0.626 (0.471-0.833)	0.001^a^	0.596 (0.414-0.858)	0.005^a^	0.563 (0.373-0.849)	0.006^a^	0.723 (0.501-1.043)	0.083
Model 3	0.590 (0.438-0.795)	<0.001^a^	0.577 (0.395-0.841)	0.004^a^	0.536 (0.349-0.823)	0.004^a^	0.693 (0.474-1.013)	0.058

The unweighted multivariable Cox was adjusted for age, sex, hospital frailty level, AMI type, Killip class, hypertension, diabetes mellitus, atrial fibrillation, cerebrovascular disease, peripheral artery disease, aspirin, P2Y12 inhibitors, MRAs, SGLT2i, RASi, CCBs, and statins, without application of IPTW. IPTW models 1 to 3 incorporated additional covariate adjustment as specified below. Model 1: adjusted for age, sex, hospital frailty level, AMI types, and Killip class. Model 2: adjusted for model 1 plus comorbidities. Model 3: adjusted for model 2 plus medication use.

aHR = adjusted HR; IPTW = inverse probability of treatment weighting; other abbreviations as in Table 1.

a For *P* < 0.05.

**Table 3:** Fine-Gray Competing Risk Analyses for 1-Year Primary Composite Outcome and Heart Failure–Related Rehospitalization

1-Year Outcomes	Model 1		Model 2			Model 3
	S-HR (95% CI)	*P* Value	S-HR (95% CI)	*P* Value	S-HR (95% CI)	*P* Value
Post-AMI HFpEF						
Primary outcome	0.831 (0.648-1.060)	0.140	0.828 (0.646-1.060)	0.140	1.020 (0.779-1.330)	0.910
HF-related rehospitalization	1.080 (0.776-1.501)	0.660	1.060 (0.766-1.480)	0.710	1.010 (0.721-1.420)	0.950
Post-AMI HFrEF/HFmrEF						
Primary outcome	0.631 (0.495-0.804)	<0.001^a^	0.628 (0.492-0.802)	<0.001^a^	0.628 (0.484-0.815)	<0.001^a^
HF-related rehospitalization	0.972 (0.685-1.380)	0.870	0.964 (0.678-1.370)	0.840	0.796 (0.533-1.150)	0.220

All Fine-Gray models were estimated within the IPTW pseudopopulation with additional covariate adjustment as specified in models 1 to 3, consistent with the Cox proportional hazards analyses reported in Table 2. Model 1: adjusted for age, sex, hospital frailty risk level, AMI types, and Killip class. Model 2: adjusted for model 1 plus comorbidities. Model 3: adjusted for model 2 plus medication use.

HF = heart failure; S-HR = subhazard HR; other abbreviations as in Table 1.

a For *P* < 0.05.

### Post-AMI HFrEF/HFmrEF

After IPTW adjustment, in patients with AMI and HFrEF/HFmrEF, in-hospital use of BBs was associated with a significantly lower incidence of 1-year primary outcome and secondary outcomes compared with those not receiving BBs (Figures 1E to 1H). Multivariable Cox regression analysis before weighting showed that in-hospital BBs use was associated with a lower risk of 1-year primary outcomes (aHR: 0.619; 95% CI: 0.483-0.792; *P* < 0.001), all-cause death (aHR: 0.596; 95% CI: 0.431-0.826; *P* = 0.002), cardiac death (aHR: 0.580; 95% CI: 0.404-0.833; *P* = 0.003), and HF-related rehospitalization (aHR: 0.725; 95% CI: 0.527-0.998; *P* = 0.049). Similarly, 3 weighted multivariable Cox models confirmed the association between BB use and reduced risk of 1-year primary outcome, all-cause mortality, and cardiac death, with no significant reduction in HF-related rehospitalization (Table 2). After IPTW, competing risk regression showed that in-hospital BBs use remained significantly associated with reduced risk of 1-year primary outcome across all 3 models (model 1 sHR: 0.631 [0.495-0.804]; model 2 sHR: 0.628 [0.492-0.802]; model 3 sHR: 0.628 [0.484-0.815], all *P* < 0.001) (Table 3). The negative control outcome of bleeding events did not differ between the 2 groups in both populations (Supplemental Table 7).

### Restricted cubic spline analysis

**Figure 2:**
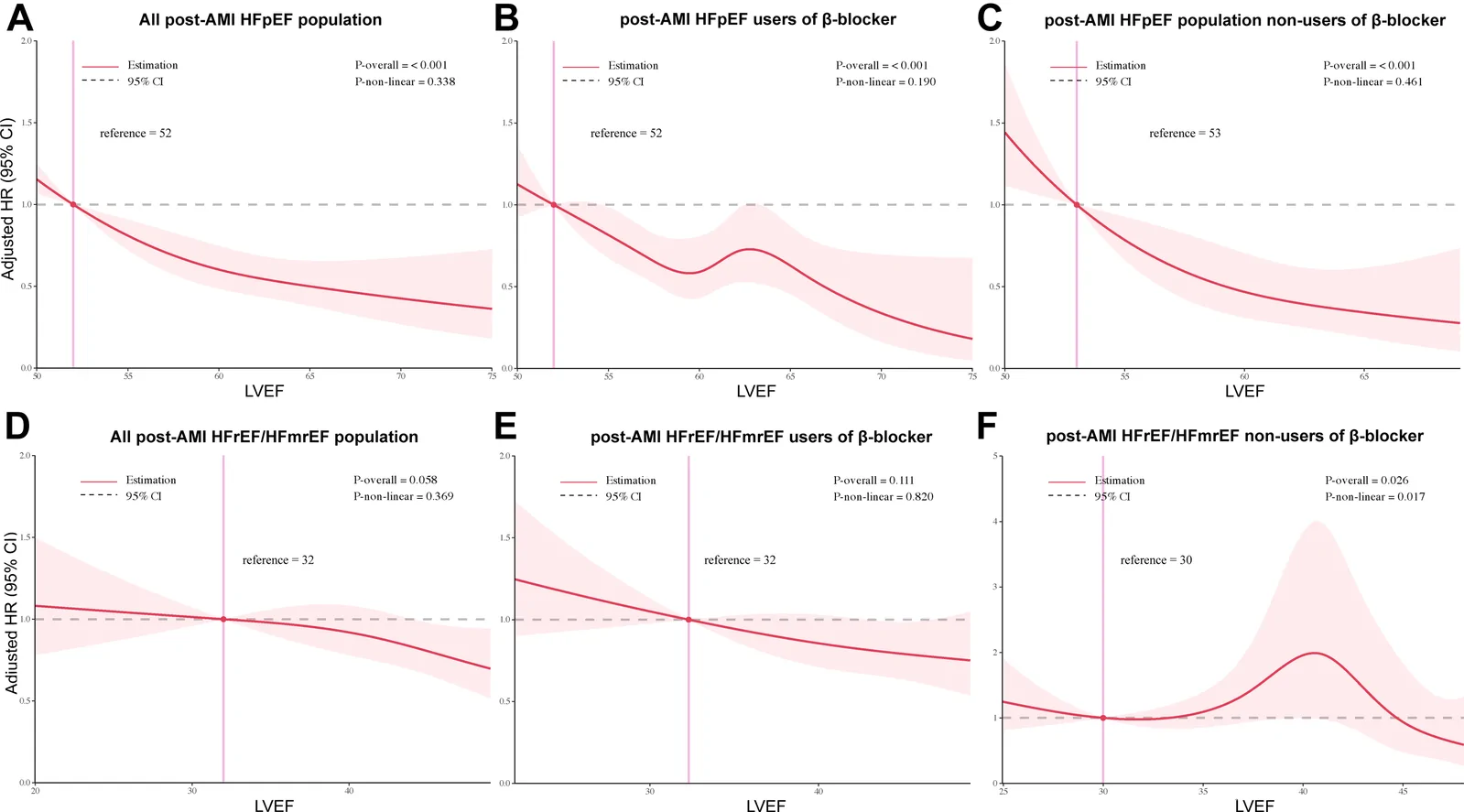
Restricted Cubic Spline Analysis of Left Ventricular Ejection Fraction and Primary Outcome (A) Overall post-AMI HFpEF cohort; (B) post-AMI HFpEF patients who received in-hospital BB therapy; (C) post-AMI HFpEF patients who did not receive in-hospital BB therapy; (D) overall post-AMI HFrEF/HFmrEF cohort; (E) post-AMI HFrEF/HFmrEF patients who received in-hospital BB therapy; and (F) post-AMI HFrEF/HFmrEF patients who did not receive in-hospital BB therapy. Solid red lines represent adjusted HRs, shaded areas represent 95% CIs, dashed horizontal lines indicate an HR of 1.0, and vertical lines mark the estimated inflection points. Multivariate restricted cubic spline regression analysis for the nonlinear association between left ventricular ejection fraction and the composite outcome of cardiac death and heart failure–related hospitalization. The restricted cubic spline models were adjusted for age, sex, acute myocardial infarction types, Killip class, aspirin, P2Y12 inhibitors, statins, calcium-channel blockers, renin-angiotensin inhibitors, mineralocorticoid receptor antagonists, sodium glucose co-transporter 2 inhibitors, peripheral vascular disease, cerebrovascular disease, diabetes mellitus, atrial fibrillation, hypertension, hospital frailty level. AMI = acute myocardial infarction; HFmrEF = heart failure with mildly reduced ejection fraction; HFpEF = heart failure with preserved ejection fraction; HFrEF = heart failure with reduced ejection fraction; LVEF = left ventricular ejection fraction; other abbreviation as in Figure 1.

RCS analyses were performed to assess the association between LVEF and 1-year primary outcome in both HFpEF and HFrEF/HFmrEF populations, stratified by BB use. In the overall post-AMI HFpEF cohort, a linear inverse association was observed between LVEF and primary outcome risk (*P*-overall <0.001, *P* nonlinear = 0.338), with an inflection LVEF at 52% (Figure 2A). This linear trend remained consistent among patients with (Figure 2B) and without BB use (Figure 2C), with respective inflection points at 52% and 53%, suggesting BB use did not significantly modify the LVEF-outcome relationship. In patients with HFrEF/HFmrEF, LVEF was linearly associated with the primary outcome in the overall population (*P* overall = 0.058, *P* nonlinear = 0.369). In no BBs group, the association was nonlinear (*P* overall = 0.026, *P* non-linear = 0.017), showing 2 inflection points and an early increase in risk when LVEF fell below 45% (Figure 2F). In contrast, this nonlinear association was not observed in HFrEF/HFmrEF patients using BBs, with a single inflection at LVEF <32% (Figure 2E). These findings suggest that the LVEF–outcome association differed according to in-hospital BB use in the HFrEF/HFmrEF population.

### Subgroup and sensitivity analysis

After adjustment using IPTW, there was no significant heterogeneity observed across subgroups for the primary or other secondary outcomes in both post-AMI HFpEF and post-AMI HFrEF/HFmrEF population (all *P* for interaction >0.05) (Supplemental Figures 4 and 5). Furthermore, considering the unique clinical characteristics of HFmrEF, an exploratory analysis stratified the post-AMI HFrEF/HFmrEF population into 2 subgroups based on LVEF (<40% and 40% to 49%). The results showed directionally consistent associations across both strata. Formal interaction testing confirmed no significant effect modification by LVEF category for both primary and secondary outcomes (all *P* for interaction >0.05) (Supplemental Table 4).

Sensitivity analyses were performed to test the robustness of findings. First, we conducted Cox regressions adjusted for the PS in both the post-AMI HFpEF and HFrEF/HFmrEF populations. Results remained consistent: BB use was not significantly associated with a 1-year primary outcome in the HFpEF population, whereas a significant association with low risk was observed in the post-AMI HFrEF/HFmrEF population (Supplemental Table 7). Second, after 1:1 PSM in 2 populations, baseline characteristics were largely balanced after matching (Supplemental Tables 5 and 6). In the matched HFpEF group, BB use remained unassociated with the 1-year primary outcome in both univariable and multivariable Cox models (Supplemental Table 7). In contrast, BB use in the matched post-AMI HFrEF/HFmrEF group was consistently associated with lower risk of 1-year primary outcome (Supplemental Table 7). We further performed win ratio analysis for the primary outcome in 2 matched populations, with cardiac death prioritized over HF-related rehospitalization. In the post-AMI HFpEF cohort, BB use yielded a win ratio of 1.053 (95% CI: 0.773-1.434; *P* = 0.744), indicating no significant advantage over the no-BB group (Supplemental Figure 6). In contrast, in the post-AMI HFrEF/HFmrEF cohort, BB use was associated with a win ratio of 1.798 (95% CI: 1.265-2.554; *P* = 0.001), demonstrating a significant advantage compared with no BB use (Supplemental Figure 7), consistent with the main analysis findings. Finally, time-stratified analyses by study period (2012-2017 vs 2018-2023) yielded consistent results: BB use remained nonsignificant in the HFpEF population, whereas it was associated with reduced 1-year primary outcome risk in the post-AMI HFrEF/HFmrEF population across both periods (Supplemental Table 7). Preadmission BB use did not differ significantly between groups before or after weighting in either HF phenotype. After excluding patients with documented preadmission BB use, the results remained consistent with the primary analysis (Supplemental Table 8).

## Discussion

In this multicenter real-world cohort, in-hospital BB use was associated with lower 1-year risks of the composite outcome, cardiac death, and all-cause death among patients with AMI and HFrEF/HFmrEF, whereas no significant association was observed among patients with AMI and preserved EF (Central Illustration). These associations remained robust after accounting for competing risks of noncardiac death and across multiple sensitivity analyses, including PSM, PS-adjusted Cox models, win ratio analysis, and time-stratified analyses. Furthermore, BBs appeared to attenuate the adverse prognostic impact of lower LVEF on the composite outcome of cardiac death and HF-related hospitalization in post-AMI HFrEF and HFmrEF patients, whereas no such effect was evident in HFpEF.

**Central Illustration:**
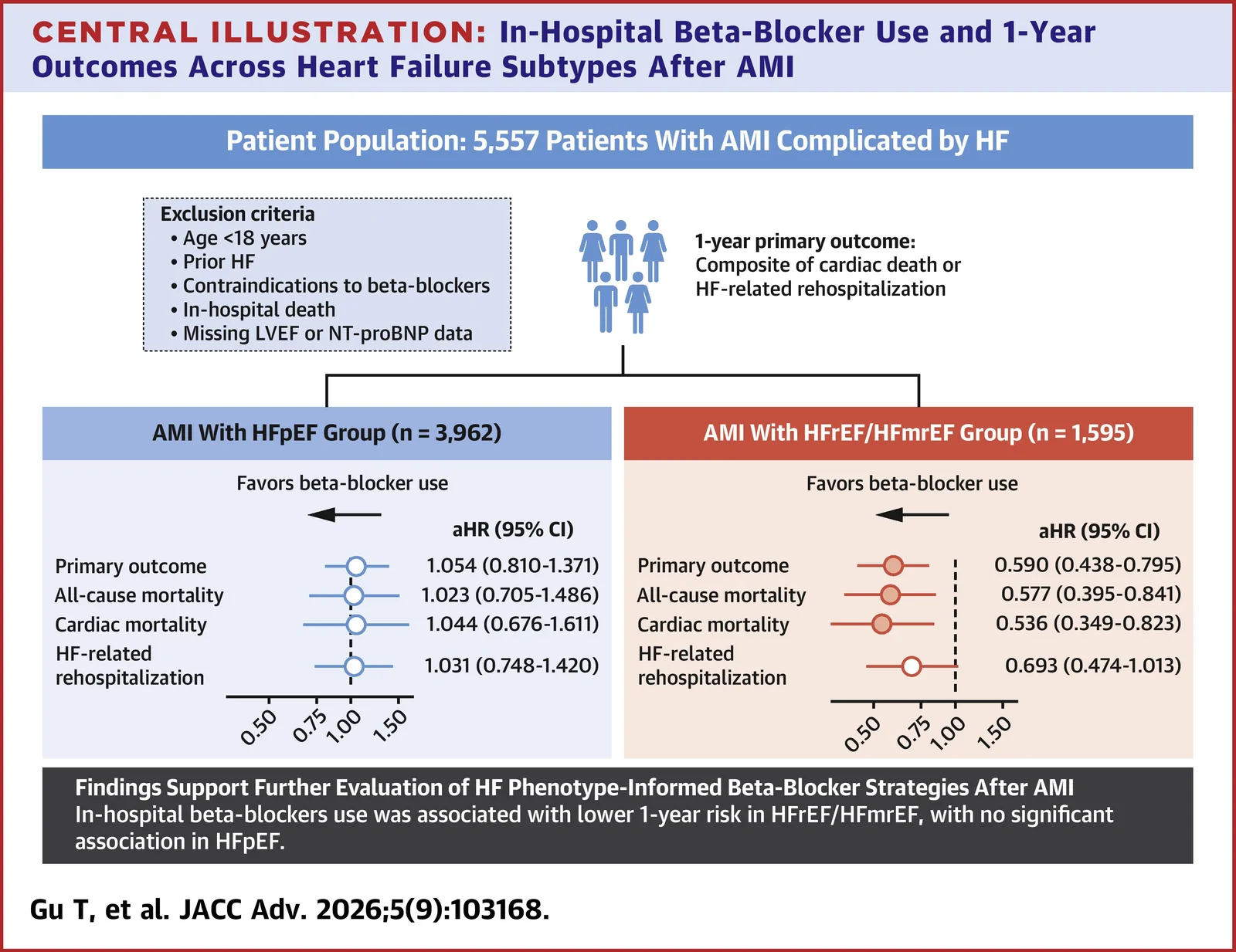
In-Hospital Beta-Blocker Use and 1-Year Outcomes Across Heart Failure Subtypes After AMI Among real-world patients with heart failure after acute myocardial infarction, in-hospital beta-blocker therapy was associated with lower 1-year risks of adverse outcomes in heart failure with reduced ejection fraction/heart failure with mildly reduced ejection fraction, whereas no significant association was observed in heart failure with preserved ejection fraction. These findings support further evaluation of heart failure phenotype-informed beta-blocker strategies after acute myocardial infarction. Abbreviations as in Figure 2.

Current guidelines continue to recommend early (<24 hours) initiation of oral BBs in ACS patients without contraindications to reduce the risk of reinfarction and ventricular arrhythmias.^6^ In our study, the high prevalence of BB initiation (∼84%) aligns with reports from major contemporary registries and trials.^23^, ^24^, ^25^, ^26^ This widespread application underscores the necessity of evaluating clinical effectiveness across distinct HF phenotypes to facilitate individualized therapy and prevent potential overtreatment in subgroups where the benefit remains unproven. However, the optimal BB strategy for different HF subtypes in AMI patients remains uncertain.^7^ Much of the evidence supporting BBs in ACS originates from the pre-reperfusion era.^27^, ^28^, ^29^ A meta-analysis of 60 ACS randomized trials demonstrated a significant interaction between reperfusion status and BB efficacy, with benefits on 1-year cardiovascular events observed only in the pre-reperfusion era and no mortality benefit in the reperfusion era.^30^ That analysis, however, did not stratify by HF phenotype, precluding conclusions regarding differential treatment effects in HFpEF vs HFrEF/HFmrEF after AMI. More recent contemporary analyses, such as a meta-analysis of 290,349 post-AMI patients without reduced LVEF or a history of HF, found no significant effect of BBs on all-cause or cardiac mortality in the modern reperfusion era,^31^ consistent with our findings in post-AMI HFpEF. Notably, this meta-analysis also identified 2010 as a pivotal inflection point separating the pre- and post-reperfusion eras—a time frame that coincides with the period covered in our study—further supporting the temporal and clinical relevance of our findings. Importantly, our study population was defined by the coexistence of both LVEF status and clinical HF symptoms, rather than either criterion alone, which may better reflect patient subgroups with distinct prognoses and therapeutic needs.^32^

We classified patients using an LVEF threshold of 50%, grouping HFmrEF and HFrEF together. Although HFmrEF shares some intermediate characteristics with HFpEF, it also exhibits substantial similarities with HFrEF in comorbidity burden, risk factor profiles, and therapeutic responses.^15^^,^^16^^,^^33^ Recently, an individual patient data meta-analysis by Rossello et al^34^ pooled data from 4 contemporary trials, demonstrating that BB therapy significantly reduced the composite risk of death, recurrent MI, or HF in post-AMI patients with mildly reduced LVEF (40% to 49%). Their study established the foundational efficacy of BBs in this LVEF spectrum. Our study further provides real-world evidence supporting these benefits in post-AMI patients with clinically evident HFmrEF and HFrEF. Recent European Society of Cardiology ACS guidelines recommend BBs for both HFmrEF and HFrEF after AMI, but not for HFpEF.^7^ Notably, HFpEF accounts for a large proportion of HF complicating AMI—71.3% in our cohort—highlighting the need for evidence-based strategies in this population.

Several contemporary studies are aligned with our results. Song et al stratified AMI patients by an LVEF threshold of 50% and found that BBs conferred no prognostic benefit in those with LVEF ≥50%, consistent with our HFpEF findings.^35^ However, their classification relied solely on LVEF and did not require the presence of HF evidence. Similarly, Ishak et al^36^ reported in a nationwide cohort that BB therapy beyond 1-year post-AMI was not associated with improved cardiovascular outcomes in patients without HF or left ventricular systolic dysfunction. In contrast, Kim et al^37^ observed a mortality benefit from 1-year BB therapy in post-AMI patients without clinical HF, but their cohort did not restrict LVEF, likely including asymptomatic individuals with reduced or mildly reduced LVEF—introducing potential bias. Indeed, the CAPITAL-RCT (Carvedilol Post-Intervention Long-Term Administration in Large-Scale Randomized Controlled Trial), an earlier RCT evaluating carvedilol in the primary PCI era, had already demonstrated no significant clinical benefit for uncomplicated STEMI patients.^38^ More recently, 4 recent landmark RCTs evaluating BBs in post-AMI patients without clinical HF have yielded a spectrum of foundational insights. The pooled analysis of the BETAMI (Beta-Blocker Treatment After Acute Myocardial Infarction in Patients Without Reduced Left Ventricular Systolic Function) and the DANBLOCK (Danish Trial of Beta-Blocker Treatment After Myocardial Infarction Without Reduced Ejection Fraction), referred to as BETAMI-DANBLOCK, suggested a modest prognostic benefit for BB initiation (HR: 0.85; 95% CI: 0.75-0.98).^39^ In contrast, the REBOOT-CNIC (Treatment with Beta-Blockers after Myocardial Infarction without Reduced Ejection Fraction) trial (HR: 1.04; 95% CI: 0.89-1.22) and REDUCE-AMI demonstrated neutral outcomes.^8^^,^^40^ Furthermore, the ABYSS trial, which focused on treatment duration rather than initiation, found that interrupting BB therapy in patients with LVEF ≥40% was not noninferior to continuation.^9^ The enrollment criteria and near-universal optimal revascularization (eg, >95% PCI rate in REDUCE-AMI) in these RCTs reflect rigorous, highly controlled study designs perfectly aligned with their specific research questions. Whereas the aforementioned trials primarily enrolled patients based on specific LVEF thresholds, our study required the concurrent presence of documented HF symptoms or biomarker evidence of congestion alongside LVEF classification. This dual-criterion approach is clinically important, as evidence suggests that AMI patients exhibiting both LVEF changes and clinical HF symptoms face a distinctly different prognosis compared to those with isolated LVEF reductions.^32^ Ultimately, our findings complement the existing randomized evidence by extending the evaluation of BB effectiveness to a more heterogeneous, high-risk real-world population, thereby facilitating more tailored post-AMI care strategies.

The differential effects of BBs among HF phenotypes with varying LVEFs after AMI are biologically plausible. Beyond their anti-ischemic effects, BBs reduce sympathetic overactivation, lower heart rate, prevent calcium overload and left ventricular hypertrophy, decrease afterload, and suppress ventricular arrhythmias, all of which may contribute to improved outcomes in HFmrEF and HFrEF after AMI.^16^^,^^35^ In contrast, advances in AMI management—including primary PCI, fibrinolysis, potent antiplatelet therapy, and physiologic lesion assessment—have reduced infarct size and attenuated sympathetic activation, potentially diminishing the need for β-blockade in patients without significant myocardial injury.^41^^,^^42^ Rapid reperfusion itself strongly suppresses sympathetic activity, raising questions about the incremental benefit of BBs in fully revascularized patients without LV systolic dysfunction. The RCS analysis suggested a less adverse LVEF–outcome relationship among BB-treated patients with HFrEF or HFmrEF, although this finding should be interpreted as exploratory. It is also important to recognize that HFpEF in the AMI setting is pathophysiologically distinct from HFrEF/HFmrEF. Whereas HFrEF/HFmrEF after AMI is largely a direct consequence of acute myocyte loss and left ventricular systolic remodeling, HF with preserved EF in this context most often represents acute decompensation or clinical unmasking of a pre-existing—and frequently subclinical—diastolic or structural substrate (such as hypertensive heart disease, left ventricular hypertrophy, or aging), precipitated by the acute ischemic and hemodynamic stress of infarction, rather than a new HFpEF entity created by the infarct itself.^14^ However, BBs may be less beneficial in patients with AMI and HFpEF; by prolonging diastolic filling time, they can increase left ventricular volume and pressure, potentially worsening congestion in patients with impaired diastolic reserve.^25^ Chronotropic incompetence is common in HFpEF, and pharmacologic heart rate reduction may impair functional capacity.^43^ Observational studies have associated BBs with higher risks of HF hospitalization in patients with LVEF ≥40% and, in a secondary analysis of the TOPCAT (Treatment of Preserved Cardiac Function Heart Failure with an Aldosterone Antagonist) trial, in those with EF >50%.^44^, ^45^, ^46^ Furthermore, BBs are linked to several adverse effects—including depression and fatigue—that can negatively impact quality of life, leading to poor adherence or discontinuation.^47^ Discontinuation of BBs has been shown to improve functional capacity in HFpEF patients with dynamic systolic dysfunction and may enhance their response to exercise training.^48^ In our population with AMI and preserved EF, we observed neither a clear prognostic benefit nor evidence of harm from BB therapy. Further large-scale trials and mechanistic studies, ideally incorporating diastolic-function phenotyping, are warranted to clarify the safety and potential role of BBs in AMI complicated by HF with preserved EF.

### Study Limitations

Several limitations should also be acknowledged. First, as an observational study, residual confounding and selection bias cannot be completely excluded despite robust adjustment. Second, although we reported preadmission BB use and performed a sensitivity analysis excluding patients with documented preadmission BB therapy, we could not systematically track BB dose titration or long-term postdischarge adherence because of database limitations. Therefore, the exposure definition may not fully capture precise long-term treatment duration or adherence, and the observed 1-year associations should not be interpreted as causal effects of sustained BB therapy. Third, patients receiving BBs had more favorable baseline profiles, although we used IPTW, multivariable adjustment, PS adjustment, PSM, competing risk models, and negative-control analysis; residual confounding by indication and unmeasured disease severity cannot be excluded. Fourth, outcomes were limited to 1-year follow-up, and longer-term effects remain to be clarified. Fifth, LVEF-based HF phenotyping was based on echocardiography performed during the index hospitalization, and systematic follow-up LVEF data were not available; therefore, we could not assess LVEF recovery, deterioration, or transitions between HF phenotypes after discharge. Moreover, although patients with documented prior HF were excluded, we could not fully characterize pre-existing diastolic dysfunction or structural heart disease; therefore, some patients classified as HFpEF may have had pre-existing myocardial vulnerability unmasked or worsened by AMI. Sixth, although our study covered a large time span and multiple hospitals, the availability of echocardiographic and imaging data in the registry was limited, which reduced the final eligible sample size. This reflects an inherent limitation of real-world multicenter data sets and may affect the generalizability of the findings. Finally, external validation in other populations and prospective trials are warranted to confirm the present findings.

## Conclusions

In this multicenter cohort of patients with AMI and HF, in-hospital BBs use was associated with lower 1-year mortality and adverse cardiovascular outcomes among those with HFrEF/HFmrEF but not among those with HFpEF. These findings should be interpreted as associations and support further evaluation of HF phenotype-informed secondary prevention strategies after AMI. The neutral association observed in the HFpEF phenotype should be interpreted within the context of AMI patients with HF evidence and preserved EF during index hospitalization, rather than generalized to all patients with established chronic HFpEF after AMI.

Perspectives **COMPETENCY IN MEDICAL KNOWLEDGE:** This study provides real-world evidence that in-hospital BB use is associated with improved 1-year outcomes in patients with AMI complicated by HFrEF/HFmrEF but not in those with HFpEF. Clinicians should recognize that HF with preserved EF in the AMI setting is pathophysiologically heterogeneous and may represent unmasking of pre-existing diastolic or structural disease rather than a direct consequence of the infarction. The absence of observed benefit in this group underscores the need for caution before extrapolating guideline recommendations derived primarily from patients with reduced EF to the broader post-AMI HF population.

**TRANSLATIONAL OUTLOOK:** Future trials should incorporate detailed diastolic-function phenotyping, serial echocardiographic follow-up, and long-term medication adherence data to clarify whether the neutral association observed in preserved-EF patients reflects a true absence of benefit or heterogeneity within this group. Additionally, integration of biomarker-guided and imaging-guided approaches may help identify subpopulations within post-AMI HFpEF who might benefit from or be harmed by BB therapy, ultimately enabling more precise, phenotype-informed treatment strategies.

## Funding support and author disclosures

This research was supported by the National Science and Technology Innovation 2030, Noncommunicable Chronic Diseases-National Science and Technology Major Project (Grant No. 2024ZD0524300, 2024ZD0524301), Tianjin Municipal Key Research Project in Priority Areas of Traditional Chinese Medicine (2022011), 10.13039/501100001809National Natural Science Foundation of China (82470527), Science and Technology Project of Tianjin Municipal Health Committee (TJWJ2024RC004), and the Key Science and Technology Support Project of Tianjin Science and Technology Bureau (24ZXGZSY00130), Key Research Project of Tianjin Education Commission (2024ZD031), Tianjin Key Medical Discipline (Specialty) Construction Project (TJYXZDXK-3-006B). Dr Lip serves as an Associate Editor and Editorial Board member of this journal; was recused from, and had no involvement in, the editorial handling or peer-review process of this article; reports institutional consultancy and/or speaker roles for BMS/Pfizer, Boehringer Ingelheim, Anthos, Huawei (no fees are received personally); and is co-PI of the AFFIRMO project on multimorbidity in AF (grant agreement No 899871), the TARGET project on digital twins for personalized management of atrial fibrillation and stroke (grant agreement No 101136244), and the ARISTOTELES project on artificial intelligence for management of chronic long-term conditions (grant agreement No 101080189), which are all funded by the EU’s Horizon Europe Research & Innovation programme. All other authors have reported that they have no relationships relevant to the contents of this paper to disclose.
