# Supplementary material for: Beta-Blocker Use After Acute Myocardial Infarction: Differential Impact Across Heart Failure Subtypes in a Real-World Cohort
Source: JACC Adv. 2026 Aug 19;5(9):103168. doi: 10.1016/j.jacadv.2026.103168 (PMC13520734; doi:10.1016/j.jacadv.2026.103168)
Supplement: Supplementary file 1 [file mmc1.docx]

**β-Blocker use After Acute Myocardial Infarction: Differential Impact Across Heart Failure Subtypes in Real-World Cohort**

**Supplementary Online Content**

**eTable1.** ICD-10 codes used to identify comorbidities from the Tianjin Coronary Artery Disease Specialized Database

| **Diagnoses** | **ICD-10 Code** |
| --- | --- |
| Peripheral Vascular Disease | I70.x, I71.x, I731.x, I738.x, I739.x, I771.x, I790.x, I792.x, K551.x, K558.x, K559.x, Z958.x, Z959.x |
| Cerebrovascular Disease | G45x, G46x, H340x, I61x, I62x, I63x, I64x, I65x, I66x, I67x, I68x, I69x |
| Diabetes Mellitus | E10.x, E11.x, E12.x, E13.x, E14.x |
| Hypertension | I10.x, I11.x, I13.x, I14.x, I15.x |
| Atrial Fibrillation | I48.x |
| Cancer | C.x |
| Bleeding Events (including gastrointestinal, intracranial, ocular, respiratory, hemopericardium and hemarthrosis bleeding) | K25.0, K25.1, K25.2, K25.4, K25.5, K25.6, K26.0, K26.1, K26.2, K26.4, K26.5, K26.6, K28.0, K28.1, K28.2, K28.4, K28.5, K28.6, K27.0, K27.1, K27.2, K27.4, K27.5, K27.6, K31.82, K55.22, K22.6, K29.0, K29.21, K29.31, K29.41, K29.51, K29.61, K29.81, K29.71, K29.91, K62.5 I85.01, I85.11, K92.xx, I60, I61, I62, I69.1, I69.2, H35.6, H43.1, R04, I31.2, K66.1, M25.0 |
| Myocardial Infarct | I21.xx, I22.xx, I21.4x |

ICD-10: 10^th^ revision of the International Classification of Diseases system.

**eTable2.** Baseline characteristics of patients with and without missing baseline NT-proBNP data.

| **Characteristics** | **NT-proBNP available** **(n = 5557)** | **NT-proBNP missing** **(n = 37)** | **P value** |
| --- | --- | --- | --- |
| Male, n (%) | 3763 (67.7) | 28 (75.7) | 0.392 |
| Age, mean (SD) | 66.24 (12.07) | 65.05 (10.64) | 0.552 |
| STEMI, n (%) | 2605 (46.9) | 16 (43.2) | 0.782 |
| Killip class, n (%) |  |  | 0.172 |
| I | 1732 (31.2) | 8 (21.6) |  |
| II | 2868 (51.6) | 19 (51.4) |  |
| III | 719 (12.9) | 6 (16.2) |  |
| IV | 238 (4.3) | 4 (10.8) |  |
| Hospital frailty risk level, n (%) |  |  | 0.120 |
| Low risk | 3295 (59.3) | 28 (75.7) |  |
| Intermediate | 2203 (39.6) | 9 (24.3) |  |
| High risk | 59 (1.1) | 0 (0.0) |  |
| LVEF, mean (SD) | 53.95 (9.60) | 55.92 (9.42) | 0.213 |
| *Comorbidities* |  |  |  |
| Peripheral Vascular Disease, n (%) | 518 (9.3) | 4 (10.8) | 0.979 |
| Cerebrovascular Disease, n (%) | 1445 (26.0) | 8 (21.6) | 0.676 |
| Gastrointestinal Bleeding, n (%) | 160 (2.9) | 1 (2.7) | 1.000 |
| Diabetes Mellitus, n (%) | 2102 (37.8) | 12 (32.4) | 0.614 |
| Malignancy, n (%) | 142 (2.6) | 0 (0.0) | 0.645 |
| Atrial Fibrillation, n (%) | 702 (12.6) | 4 (10.8) | 0.933 |
| Hypertension, n (%) | 3570 (64.2) | 20 (54.1) | 0.264 |
| *Medications* |  |  |  |
| PCI, n (%) | 3356 (60.4) | 27 (73.0) | 0.164 |
| Primary PCI, n (%) | 2663 (47.9) | 24 (64.9) | 0.059 |
| Aspirin, n (%) | 5143 (92.5) | 36 (97.3) | 0.433 |
| P2Y12 inhibitors, n (%) | 5361 (96.5) | 37 (100.0) | 0.475 |
| Statins, n (%) | 5369 (96.6) | 37 (100.0) | 0.496 |
| CCBs, n (%) | 987 (17.8) | 7 (18.9) | 1.000 |
| MRAs, n (%) | 3094 (55.7) | 23 (62.2) | 0.532 |
| RASis, n (%) | 4543 (81.8) | 32 (86.5) | 0.596 |
| SGLT2 inhibitors, n (%) | 464 (8.3) | 2 (5.4) | 0.728 |
| Loop diuretics, n (%) | 4092 (73.6) | 28 (75.7) | 0.926 |
| OACs, n (%) | 166 (3.0) | 1 (2.7) | 1.000 |
| Pre-admission BB use, n (%) | 1822 (32.8) | 7 (18.9) | 0.106 |

Data are presented as mean (SD) or n (%). Groups were compared using the t-test for continuous variables and the χ² test or Fisher's exact test for categorical variables, as appropriate. Abbreviations: BB: beta-blocker; CCBs: calcium channel blockers; LVEF: left ventricular ejection fraction; MRAs: aldosterone receptor antagonists; NT-proBNP: N-terminal pro-B-type natriuretic peptide; OACs: oral anticoagulants; PCI: percutaneous coronary intervention; RASis: renin-angiotensin system inhibitors; SD: standard deviation; SGLT2: sodium-glucose cotransporter 2; STEMI: ST-elevation myocardial infarction.

**eTable3.** Negative control analysis (1-year Bleeding events).

| ***Negative outcome***  ***analysis*** | 1-year Bleeding events | |
| --- | --- | --- |
| ***Post-AMI HFpEF*** | aHR (95% CI) \| P value | |
| **Unweighted multivariable Cox** | 1.907 (0.631 -3.221) | 0.218 |
| **IPTW models with additional covariate adjustment** |  |  |
| Model 1 | 1.455 (0.813-2.605) | 0.206 |
| Model 2 | 1.436 (0.801-2.574) | 0.223 |
| Model 3 | 1.445 (0.813-2.569) | 0.209 |
| ***Post-AMI HFmrEF & HFrEF*** |  |  |
| **Unweighted multivariable Cox model** | 0.825 (0.428-1.591) | 0.565 |
| **IPTW models with additional covariate adjustment** |  |  |
| Model 1 | 0.761 (0.379 -1.529) | 0.444 |
| Model 2 | 0.792 (0.402-1.558) | 0.499 |
| Model 3 | 0.796 (0.401-1.581) | 0.515 |

Model 1: adjusted for age, sex, hospital frailty risk level, AMI type and Killip class.

Model 2: adjusted for Model 1 plus comorbidities.

Model 3: adjusted for Model 2 plus medication use.

Abbreviations: aHR: adjusted hazard ratio; AMI: acute myocardial infarction; CI: confidence interval.

**eTable4.** Exploratory analysis of clinical outcomes associated with beta-blocker therapy stratified by left ventricular ejection fraction (<40% vs. 40–49%)

| Outcome | Subgroup | HR (95% CI) | P value | P for interaction |
| --- | --- | --- | --- | --- |
| **Primary outcome** |  |  |  | 0.864 |
|  | HFrEF | 0.58 (0.39–0.85) | 0.006* |  |
|  | HFmrEF | 0.54 (0.32–0.89) | 0.016* |  |
| **All-cause death** |  |  |  | 0.989 |
|  | HFrEF | 0.54 (0.34–0.87) | 0.011* |  |
|  | HFmrEF | 0.43 (0.2–0.96) | 0.040* |  |
| **Cardiac death** |  |  |  | 0.902 |
|  | HFrEF | 0.52 (0.31–0.88) | 0.015* |  |
|  | HFmrEF | 0.35 (0.14–0.86) | 0.022* |  |
| **HF-related rehospitalisation** |  |  |  | 0.760 |
|  | HFrEF | 0.68 (0.42–1.11) | 0.122 |  |
|  | HFmrEF | 0.72 (0.41–1.25) | 0.239 |  |

*For P < 0.05;

Abbreviations: CI, confidence interval; HFmrEF, heart failure with mildly reduced ejection fraction; HFrEF, heart failure with reduced ejection fraction; HR, hazard ratio; LVEF, left ventricular ejection fraction.

**eTable5.** Baseline characteristics of post-AMI HFpEF population after propensity score matching.

|  | **No BBs group**  **(n = 576)** | **BBs group**  **(n = 576)** | **P after PSM** | **SMD after PSM** |
| --- | --- | --- | --- | --- |
| Male, n (%) | 364 (63.2) | 366 (63.5) | 0.951 | 0.007 |
| Age (mean (SD)) | 68.18 (11.28) | 68.38 (11.42) | 0.767 | 0.017 |
| STEMI, n (%) | 297 (51.6) | 291 (50.5) | 0.768 | 0.021 |
| PCI | 306 (53.1) | 296 (51.4) | 0.596 | 0.035 |
| Primary PCI | 253 (43.9) | 241 (41.8) | 0.513 | 0.042 |
| KILLIP, n (%) |  |  | 0.643 | 0.076 |
| I | 202 (35.1) | 219 (38.0) |  |  |
| II | 276 (47.9) | 272 (47.2) |  |  |
| III | 75 (13.0) | 66 (11.5) |  |  |
| IV | 23 (4.0) | 19 (3.3) |  |  |
| Hospital frailty risk level |  |  | 0.348 | 0.086 |
| *Low risk* | 335 (58.2) | 357 (62.0) |  |  |
| *Intermediate* | 232 (40.3) | 213 (37.0) |  |  |
| *High risk* | 9 (1.6) | 6 (1.0) |  |  |
| LVEF | 60 [56, 62] | 60 [57, 62] | 0.267 | 0.058 |
| NT-proBNP | 917.70 [367.75, 2688.75] | 1071.50 [416.80, 2589.00] | 0.261 | 0.041 |
| ***Comorbidities*** |  |  |  |  |
| CCI | 0.50 [0.00, 2.00] | 0.00 [0.00, 2.00] | 0.292 | 0.013 |
| Peripheral Vascular Disease | 76 (13.2) | 62 (10.8) | 0.238 | 0.075 |
| Cerebrovascular Disease | 157 (27.3) | 155 (26.9) | 0.947 | 0.008 |
| Gastrointestinal Bleeding | 23 (4.0) | 20 (3.5) | 0.756 | 0.027 |
| Diabetes Mellitus | 181 (31.4) | 184 (31.9) | 0.899 | 0.011 |
| Malignancy | 20 (3.5) | 23 (4.0) | 0.756 | 0.027 |
| Atrial Fibrillation | 70 (12.2) | 71 (12.3) | 1.000 | 0.005 |
| Hypertension | 373 (64.8) | 354 (61.5) | 0.272 | 0.068 |
| ***Medications*** |  |  |  |  |
| Aspirin | 501 (87.0) | 513 (89.1) | 0.318 | 0.064 |
| P2Y12 inhibitors | 536 (93.1) | 545 (94.6) | 0.327 | 0.065 |
| Statins | 540 (93.8) | 556 (96.5) | 0.040 | 0.129 |
| CCBs | 124 (21.5) | 107 (18.6) | 0.239 | 0.074 |
| MRAs | 248 (43.1) | 243 (42.2) | 0.812 | 0.018 |
| RASis | 378 (65.6) | 374 (64.9) | 0.853 | 0.015 |
| SGLT2 inhibitors | 36 (6.2) | 36 (6.2) | 1.000 | <0.001 |
| loop diuretics | 357 (62.0) | 348 (60.4) | 0.629 | 0.032 |
| OACs | 21 (3.6) | 20 (3.5) | 1.000 | 0.009 |

Abbreviations: AMI: acute myocardial infarction; CCBs: calcium channel blockers; HFpEF: heart failure with preserved ejection fraction; LVEF: left ventricular ejection fraction; MRAs: aldosterone receptor antagonists; NT-proBNP: N-terminal pro-B-type natriuretic peptide; OACs: oral anticoagulants; PCI: percutaneous coronary intervention; RAS: renin-angiotensin-system; SGLT2i, sodium-glucose cotransporter 2 inhibitors; SD: standard deviation; SMD: standardized mean difference; STEMI: ST-elevation myocardial infarction.

**eTable6.** Baseline characteristics of post-AMI HFrEF & HFmrEF population after propensity score matching.

|  | **No BBs group**  **(n = 204)** | **BBs group**  **(n = 204)** | **P after PSM** | **SMD after PSM** |
| --- | --- | --- | --- | --- |
| ***Demographics*** |  |  |  |  |
| Male, n (%) | 137 (67.2) | 144 (70.6) | 0.521 | 0.074 |
| Age (mean (SD)) | 70.78 (11.64) | 71.37 (11.12) | 0.605 | 0.051 |
| STEMI, n (%) | 94 (46.1) | 88 (43.1) | 0.618 | 0.059 |
| PCI | 80 (39.2) | 83 (40.7) | 0.840 | 0.030 |
| Primary PCI | 58 (28.4) | 62 (30.4) | 0.744 | 0.043 |
| KILLIP, n (%) |  |  | 0.970 | 0.049 |
| I | 54 (26.5) | 52 (25.5) |  |  |
| II | 81 (39.7) | 85 (41.7) |  |  |
| III | 50 (24.5) | 50 (24.5) |  |  |
| IV | 19 (9.3) | 17 (8.3) |  |  |
| Hospital frailty risk level |  |  | 0.088 | 0.220 |
| *Low risk* | 75 (36.8) | 97 (47.5) |  |  |
| *Intermediate* | 123 (60.3) | 102 (50.0) |  |  |
| *High risk* | 6 (2.9) | 5 (2.5) |  |  |
| LVEF | 41 [36, 46] | 41 [38, 45] | 0.617 | 0.080 |
| NT-proBNP | 4777.00  [1368.00, 12000.00] | 4786.50  [1443.75, 13777.50] | 0.644 | 0.104 |
| ***Comorbidities*** |  |  |  |  |
| CCI | 1.00 [0.00, 3.00] | 1.00 [0.00, 3.00] | 0.196 | 0.135 |
| Peripheral Vascular Disease | 23 (11.3) | 22 (10.8) | 1.000 | 0.016 |
| Cerebrovascular Disease | 71 (34.8) | 63 (30.9) | 0.461 | 0.084 |
| Gastrointestinal Bleeding | 9 (4.4) | 5 (2.5) | 0.415 | 0.108 |
| Diabetes Mellitus | 91 (44.6) | 78 (38.2) | 0.228 | 0.130 |
| Malignancy | 9 (4.4) | 4 (2.0) | 0.260 | 0.140 |
| Atrial Fibrillation | 40 (19.6) | 42 (20.6) | 0.902 | 0.024 |
| Hypertension | 131 (64.2) | 128 (62.7) | 0.837 | 0.031 |
| ***Medications*** |  |  |  |  |
| Aspirin | 170 (83.3) | 179 (87.7) | 0.260 | 0.126 |
| P2Y12 inhibitors | 189 (92.6) | 194 (95.1) | 0.409 | 0.102 |
| Statins | 186 (91.2) | 187 (91.7) | 1.000 | 0.018 |
| CCBs | 35 (17.2) | 29 (14.2) | 0.496 | 0.081 |
| MRAs | 138 (67.6) | 136 (66.7) | 0.916 | 0.021 |
| RASis | 124 (60.8) | 124 (60.8) | 1.000 | <0.001 |
| SGLT2 inhibitors | 23 (11.3) | 28 (13.7) | 0.549 | 0.074 |
| loop diuretics | 170 (83.3) | 171 (83.8) | 1.000 | 0.013 |
| OACs | 9 (4.4) | 13 (6.4) | 0.511 | 0.087 |

Abbreviations: ACEI: angiotensin-converting enzyme inhibitors; AMI: acute myocardial infarction; CCBs: calcium channel blockers; HFmrEF: heart failure with mildly reduced ejection fraction; HFpEF: heart failure with preserved ejection fraction; HFrEF: heart failure with reduced ejection fraction; LVEF: left ventricular ejection fraction; MRAs: aldosterone receptor antagonists; NT-proBNP: N-terminal pro-B-type natriuretic peptide; OACs: oral anticoagulants; PCI: percutaneous coronary intervention; RAS: renin-angiotensin-system; SGLT2i, sodium-glucose cotransporter 2 inhibitors; SD: standard deviation; SMD: standardized mean difference; STEMI: ST-elevation myocardial infarction.

**eTable7.** Sensitivity analysis of the primary outcome using propensity score matching and year-stratified cox analysis

| ***Methods*** | aHR (95% CI) | P value | |
| --- | --- | --- | --- |
| ***Post-AMI HFpEF*** |  | | |
| **Propensity score matching** |  | |  |
| Univariable model | 0.959 (0.715-1.286) | | 0.779 |
| Multivariable adjusted | 0.986 (0.724-1.345) | | 0.933 |
| Adjusted for propensity score | 1.024 (0.799-1.313) | | 0.849 |
| ***Year-stratified analyses*** |  | |  |
| 2012-2017 | 0.852 (0.574-1.265) | | 0.428 |
| 2018-2023 | 1.390 (0.989-1.955) | | 0.058 |
| ***Post-AMI HFmrEF & HFrEF*** |  | |  |
| **Propensity score matching** |  | |  |
| Univariable model | 0.556 (0.393-0.785) | | <0.001* |
| Multivariable adjusted | 0.481 (0.331-0.698) | | <0.001* |
| Adjusted for propensity score | 0.655 (0.509 -0.843) | | 0.001* |
| ***Year-stratified analyses*** |  | |  |
| 2012-2017 | 0.499 (0.291-0.855) | | 0.011* |
| 2018-2023 | 0.696 (0.516-0.939) | | 0.018* |

*For P < 0.05;

Cox models in the propensity score-matched cohorts were stratified by matched pair to account for the matched design. Abbreviations: aHR: adjusted hazard ratio; CI: confidence interval; HFmrEF: heart failure with mildly reduced ejection fraction; HFpEF: heart failure with preserved ejection fraction; HFrEF: heart failure with reduced ejection fraction.

**eTable 8.** Sensitivity analysis of 1-year outcomes after excluding patients with pre-admission beta-blocker use

| **Population** | **Analysis** | **1-year Primary outcome aHR (95% CI)** | **P value** | **1-year All-cause mortality aHR (95% CI)** | **P value** | **1-year Cardiac mortality aHR (95% CI)** | **P value** | **1-year HF-related rehospitalization aHR (95% CI)** | **P value** |
| --- | --- | --- | --- | --- | --- | --- | --- | --- | --- |
| HFpEF | Before IPTW | 1.11  (0.78-1.57) | 0.568 | 1.39  (0.88-2.21) | 0.159 | 1.22  (0.69-2.18) | 0.495 | 1.07  (0.70-1.65) | 0.755 |
|  | After IPTW | 1.09  (0.74-1.59) | 0.666 | 1.14  (0.70-1.86) | 0.605 | 1.04  (0.57-1.91) | 0.891 | 1.09  (0.69-1.73) | 0.710 |
| HFrEF/HFmrEF | Before IPTW | 0.40  (0.27-0.57) | <0.001* | 0.30  (0.18-0.48) | <0.001* | 0.28  (0.16-0.49) | <0.001* | 0.58  (0.35-0.96) | 0.035* |
|  | After IPTW | 0.48  (0.31-0.75) | 0.001* | 0.41  (0.24-0.69) | <0.001* | 0.39  (0.25-0.71) | 0.002* | 0.63  (0.36-1.11) | 0.111 |

*For P < 0.05.

This sensitivity analysis excluded patients with documented pre-admission beta-blocker use. The weighted analysis was performed using inverse probability of treatment weighting. The primary outcome was the composite of cardiac death and HF-related rehospitalization. **Abbreviations**: aHR, adjusted hazard ratio; AMI, acute myocardial infarction; BB, beta-blocker; CI, confidence interval; HF, heart failure; HFmrEF, heart failure with mildly reduced ejection fraction; HFpEF, heart failure with preserved ejection fraction; HFrEF, heart failure with reduced ejection fraction; IPTW, inverse probability of treatment weighting.

**eFigure1.** Flowchart of patient selection and study design.

Flowchart detailing the patient selection process, stratification into heart failure subtypes, and the statistical analysis framework.

Abbreviations: AMI: acute myocardial infarction; HF: heart failure; HFmrEF: heart failure with mildly reduced ejection fraction; HFpEF: heart failure with preserved ejection fraction; HFrEF: heart failure with reduced ejection fraction; IPTW: inverse probability of treatment weighting; LVEF: left ventricular ejection fraction; NT-proBNP: N-terminal pro-B-type natriuretic peptide; PS: propensity score; RCS: restricted cubic spline.

**eFigure2.** Directed acyclic graph of causal structure among study exposure, outcome, and confounders.

Directed acyclic graph (DAG) illustrating the assumed causal relationships between in-hospital BBs use, and the 1-year outcome (composite of cardiac death and HF-related rehospitalization), accounting for measured and unmeasured covariates.

AMI guideline recommended medications: Aspirin, P2Y12 inhibitors, Statins; HF guideline recommended medications: RASi, MRAs, SGLT2i, loop diuretics; Comorbidities: peripheral vascular disease, cerebrovascular Disease, gastrointestinal bleeding, diabetes mellitus, malignancy, atrial fibrillation, hypertension; Hospital frailty level: frailty was categorized by hospital frailty score.

Abbreviations: AMI: acute myocardial infarction; CCBs: calcium channel blockers; HF: heart failure; MRAs: aldosterone receptor antagonists; OACs: Oral anticoagulants; RAS: renin-angiotensin-system; SGLT2i, sodium-glucose cotransporter 2 inhibitors.

**eFigure3.** SMDs before and after weighting according to beta-blocker use in patients with AMI.

The left panel presents the post-AMI HFpEF population, and the right panel presents the post-AMI HFrEF/HFmrEF population. Red circles indicate SMDs before weighting and blue squares indicate SMDs after weighting. The dashed vertical line represents the threshold of SMD = 0.1, indicating negligible imbalance. Abbreviations: AMI: acute myocardial infarction; CCBs: calcium channel blockers; HFmrEF: heart failure with mildly reduced ejection fraction; HFpEF: heart failure with preserved ejection fraction; HFrEF: heart failure with reduced ejection fraction; MRAs: aldosterone receptor antagonists; NT-proBNP: N-terminal pro-B-type natriuretic peptide; PCI: percutaneous coronary intervention; RAS: renin-angiotensin-system; SGLT2i, sodium-glucose cotransporter 2 inhibitors; SMD: standardized mean difference.

**eFigure4.** Subgroup analysis of 1 year outcome events in the post-AMI HFpEF population with main effects and interaction terms: (A) composite outcome of cardiac death and HF-related rehospitalization, (B) HF-related rehospitalization, (C) cardiac death, (D) all-cause death.

Abbreviations: AMI: acute myocardial infarction; HFpEF: heart failure with preserved ejection fraction.

**eFigure5.** Subgroup analysis of 1 year outcome events in the post-AMI HFrEF & HFmrEF population with main effects and interaction terms: (A) composite outcome of cardiac death and HF-related rehospitalization, (B) HF-related rehospitalization, (C) cardiac death, (D) all-cause death.

Abbreviations: AMI: acute myocardial infarction; HFmrEF: heart failure with mildly reduced ejection fraction; HFrEF: heart failure with reduced ejection fraction.

**eFigure6.** Win ratio of composite outcome of cardiac death and HF-related rehospitalization in post-AMI HFpEF population after PSM.

Matched pairs Win Ratio for the hierarchical composite outcome analysis of cardiac death, HF-related rehospitalization in β-blocker group versus no-β-blocker group in post-AMI HFpEF population. The composite hierarchical outcome of cardiac death, HF-related rehospitalization visits was analyzed by estimating the matched win ratio by comparing every participant in the β blocker group to every participant in the no-β blocker group to determine a winner. In the post-AMI HFpEF population, the performance of the two groups was tie. Abbreviations: AMI: acute myocardial infarction; BB: β blocker ; HFpEF: heart failure with preserved ejection fraction.

**eFigure7.** Win ratio of composite outcome of cardiac death and HF-related rehospitalization in post-AMI HFrEF & HFmrEF population after PSM.

Matched pairs Win Ratio for the hierarchical composite outcome analysis of cardiac death, HF-related rehospitalization in β-blocker group versus no-β-blocker group in post-AMI HFrEF & HFmrEF population. The composite hierarchical outcome of cardiac death, HF-related rehospitalization visits was analyzed by estimating the matched win ratio by comparing every participant in the β-blocker group to every participant in the no-β blocker group to determine a winner. In the post-AMI HFrEF & HFmrEF population, β blocker group wins over no β-blocker group for the primary outcome. Abbreviations: AMI: acute myocardial infarction; BB: β-blocker; HFmrEF: heart failure with mildly reduced ejection fraction; HFrEF: heart failure with reduced ejection fraction.
